# Reperfusion and Clinical Outcomes in Acute Ischemic Stroke: Systematic Review and Meta-Analysis of the Stent-Retriever-Based, Early Window Endovascular Stroke Trials

**DOI:** 10.3389/fneur.2018.00301

**Published:** 2018-05-14

**Authors:** Nathan W. Manning, Charles D. Warne, Philip M. Meyers

**Affiliations:** ^1^Department of Radiology and Neurological Surgery, College of Physicians and Surgeons, Columbia University, New York, NY, United States; ^2^Department of Neurological Surgery, College of Physicians and Surgeons, Columbia University, New York, NY, United States; ^3^Florey Institute of Neuroscience and Mental Health, University of Melbourne, Parkville, VIC, Australia; ^4^Roche Products Ltd., Welwyn Garden City, United Kingdom

**Keywords:** stent-retriever, endovascular, stroke, thrombectomy, reperfusion, meta-analysis

## Abstract

**Objective:**

To explore the effects of reperfusion grade rates on clinical outcomes in the setting of stent-retriever-based reperfusion therapy for anterior circulation stroke in early time windows.

**Methods:**

Systematic searching of Medline and Embase databases was performed to identify stroke trials of stent-retriever-based therapy versus standard care. Mixed effects meta-regression was used to analyze the trial-level association between reperfusion rates and clinical outcomes.

**Results:**

A total of five trials met the inclusion criteria (*n* = 1,287). Rates of successful reperfusion [modified thrombolysis in cerebral ischemia grade 2b/3] demonstrated strong evidence for an association with good functional outcomes [modified Rankin scale score (mRS) 0–2] OR 1.59 (95% CI 1.16, 2.19) *p* = 0.019 and very strong evidence for an association with excellent functional outcomes (mRS 0–1) OR 2.10 (95% CI 1.46, 3.01) *p* = 0.007. In addition, there was weak evidence for an association with symptomatic intracranial hemorrhage OR 0.54 (95% CI 0.28, 1.04) *p* = 0.057 and mortality OR 0.69 (95% CI 0.69, 1.01) *p* = 0.053.

**Conclusion:**

In early, stent-retriever-based acute ischemic stroke treatment, reperfusion appears to be a major predictor of outcomes. Every 10% increase in the rates of successful reperfusion is associated with an 11% increase in the probability of achieving good and 17% increase in the probability of achieving excellent outcomes. Symptomatic intracranial hemorrhage and mortality may be decreased as reperfusion rates are improved.

## Introduction

Stent-retriever-based mechanical thrombectomy has become the standard of care for anterior circulation, large vessel occlusion strokes (1–5). Previous studies suggested marked time dependence on the likelihood of achieving good functional outcomes (6). However, in studies limited to the use of stent-retrievers, the effect of time is greatly reduced (7). Furthermore, recent stent-retriever-based trials have demonstrated a significant treatment effect out to 24 h (8, 9). The use of stent-retrievers is associated with markedly higher rates of modified thrombolysis in cerebral ischemia (mTICI) grade 2b/3 reperfusion, which has previously been noted to correlate with trial success (10).

Successful reperfusion is associated with less infarct core growth and improved functional outcomes (11). This may help explain the treatment effect of the recent trials. While the effect of time has been explored (7, 12), to date the role of reperfusion grade in the success of these trials has not been investigated. In this study, we use meta-regression analysis to explore the association between reperfusion grade rates and outcomes in the stent-retriever-based randomized controlled trials in early treatment windows.

## Materials and Methods

This systematic review and meta-analysis was conducted as per a prespecified protocol and reported in accordance with the “Preferred Reporting Items for Systematic Reviews and Meta-Analyses” (13).

### Search Strategy

A systematic search of both the MEDLINE and EMBASE databases was performed *via* Ovid up to and including November 2016 by the principal investigator.

### Study Selection

Article abstracts and where appropriate, full text articles, were reviewed by the principal investigator. Inclusion was limited to randomized controlled trials of acute ischemic stroke, whereby stent-retriever (>75% use) was compared to standard care, including but not limited to, the use of IV-tPA. Studies were excluded if insufficient information was reported to assess the prespecified associations or trial quality. Included studies were deemed to have an overall low risk of bias as per a published risk bias assessment tool (14) and in agreement with previous meta-analyses (15, 16).

### Data Extraction

Each randomized controlled trial was individually interrogated using the available published literature. Extraction of trial-level data was performed by both Nathan W. Manning and Charles D. Warne. Patient demographics and trial characteristics, including sample size, National Institute of Health Stroke Scale, risk factors, and device type(s) were collected. Reperfusion grades and rates were extracted as reported in the individual trials, as were 90-day outcome measures including dichotomized modified Rankin Scale score (mRS 0–2 and mRS 0–1), symptomatic intracerebral hemorrhage (SICH), and mortality.

### Statistical Analysis

The association between predictors (mTICI 2b/3 reperfusion) and outcomes (mRS 0–2, mRS 0–1, mortality, and SICH) was examined at the clinical trial level. Mixed effects meta-regression was used to estimate how trial-level summaries of the predictor were associated with the trial-level log-odds of each outcome. Due to the limited number of clinical trials that met inclusion criteria, it was not feasible to include multiple predictors in the same model, so only unadjusted odds ratios were derived. To aid interpretability, the predictor was scaled so that odds ratios corresponded to the change in odds associated with a clinically achievable 10% increase in mTICI 2b/3. This was also similar to one SD from the mean of mTICI 2b/3 rates across the five trials. All analyses were performed on the intention-to-treat (ITT) population, with the robustness of conclusions assessed in the per-protocol (PP) population. Each trial was weighted by the inverse of the sum of its within-trial variance and the restricted maximum likelihood estimated residual between-trial variance. SEs were adjusted *via* the Knapp–Hartung (17) method to account for uncertainty in estimating residual heterogeneity. All statistical analyses were performed in R Studio (R Studio, Version 0.99; Boston, MA, USA).

## Results

### Search Results

The initial search identified 159 publications with potential to be included in this study. There were seven duplicates leaving 152 publications, which were screened by reviewing, title and abstract individually. Eight publications underwent full text review with five selected for inclusion, EXTEND-IA, SWIFT-PRIME, ESCAPE, and REVASCAT and MR CLEAN (1–5). The three trials excluded: THERAPY, THRACE, and PISTE (18–20) did not meet the inclusion criteria of stent-retriever use as primary therapy in ≥75% of patients by intention-to-treat analysis.

### Study Characteristics

The five included trials randomized a total of 1,287 patients to receive either the control (standard care with or without IV-tPA) or intervention (standard care plus endovascular thrombectomy). Across the five trials, 634 patients were randomized to receive endovascular therapy, and 653 patients to control. Of those randomized to the intervention, 626 patients underwent endovascular therapy (PP). All studies restricted enrollment to patients with a large vessel occlusion of the anterior circulation, confirmed on non-invasive imaging. Three trials, ESCAPE, SWIFT-PRIME, and EXTEND-IA, incorporated some form of advanced imaging to select for patients likely to have favorable penumbra to ischemic core ratios (1–3). All included trials used a stent-retriever device in >75% of the intention to treat population.

### Outcomes and Complications

The five trials assessed mRS scores at 90 days. This was the primary outcome in MR CLEAN, REVASCAT, ESCAPE, and SWIFT-PRIME (2–5). The 90-day mRS was a secondary outcome in EXTEND-IA (1). Mortality rates and rates of SICH were also assessed. Good functional outcomes (mRS 0–2) were achieved in 287 (45.3%) patients in the intervention arm at 90-days. Excellent outcomes (mRS 0–1) were attained in 173 (27.2%) patients in the intervention arm. Death occurred in 97 (15.3%) patients and SICH was reported in 27 (4.3%) patients in the intervention group (Supplementary Material).

### Reperfusion Grade

Reperfusion grade was assessed by core laboratory in each of the five trials. The thrombolysis in cerebral infarction (TICI) grade was used in ESCAPE (3). The four other trials used the mTICI grade (1, 2, 4, 5). All trials classified excellent reperfusion as 2b/3.

### Reperfusion and Outcomes

**Figure 1:**
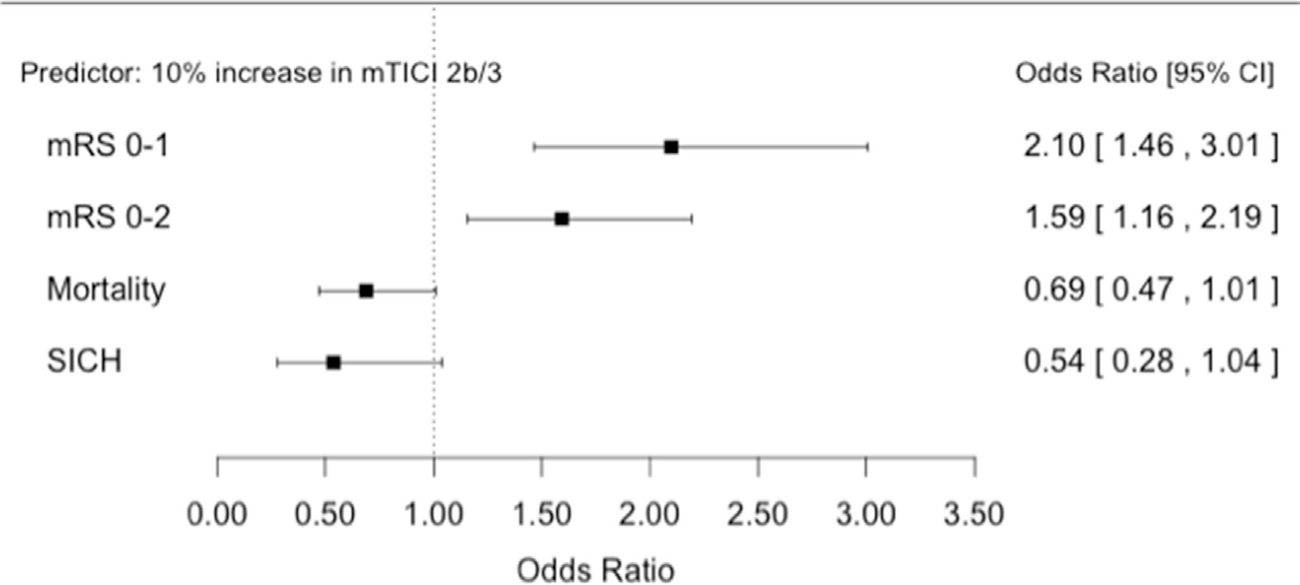
Forest plots showing odds ratios for association between successful reperfusion rates and patient outcomes.

There was evidence of an association between rates of successful reperfusion (mTICI 2b/3) and good functional outcomes OR 1.59 (95% CI 1.16, 2.19) *p* = 0.019 as well as evidence for an association with excellent outcomes OR 2.10 (95% CI 1.46, 3.01) *p* = 0.007. Reperfusion demonstrated weak evidence for an association with SICH OR 0.54 (95% CI 0.28, 1.04) *p* = 0.057 and mortality OR 0.69 (95% CI 0.47, 1.01) *p* = 0.053 (Figure 1).

**Figure 2:**
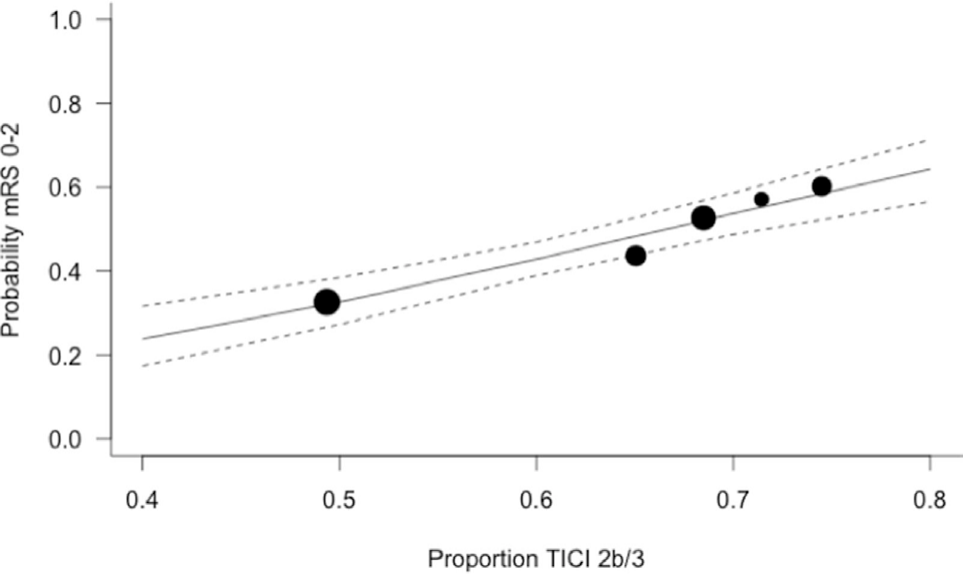
Predicted probability of modified Rankin Scale score 0–2 by modified thrombolysis in cerebral ischemia grade 2b/3, with dashed line showing 95% CI, and size of markers inversely proportional to each within-trial SD (intention-to-treat analysis).

**Figure 3:**
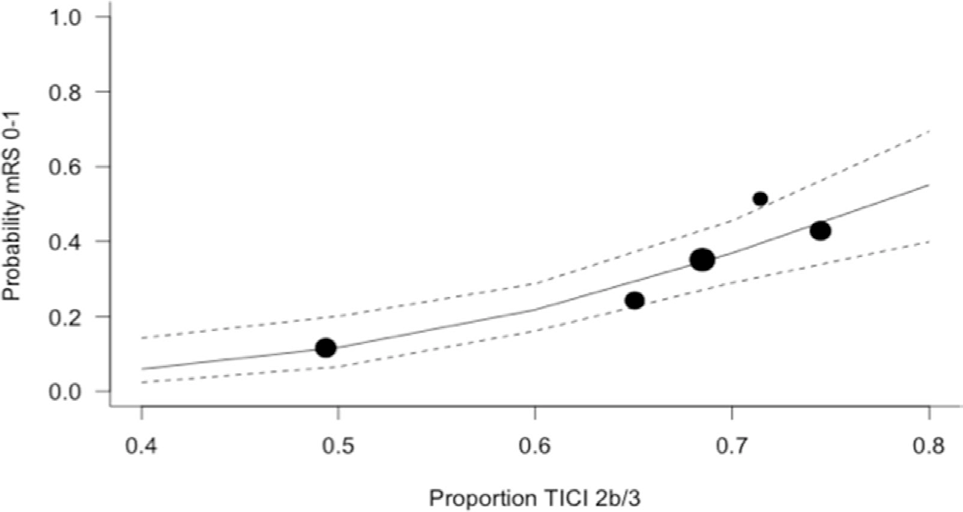
Predicted probability of modified Rankin Scale score 0–1 by modified thrombolysis in cerebral ischemia grade 2b/3, with dashed line showing 95% CI, and size of markers inversely proportional to each within-trial SD (intention-to-treat analysis).

**Figure 4:**
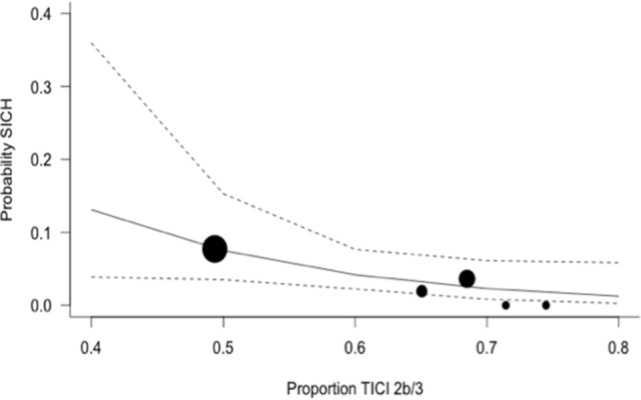
Predicted probability of symptomatic intracerebral hemorrhage (SICH) by modified thrombolysis in cerebral ischemia grade 2b/3, with dashed line showing 95% CI, and size of markers inversely proportional to each within-trial SD (intention-to-treat analysis).

**Figure 5:**
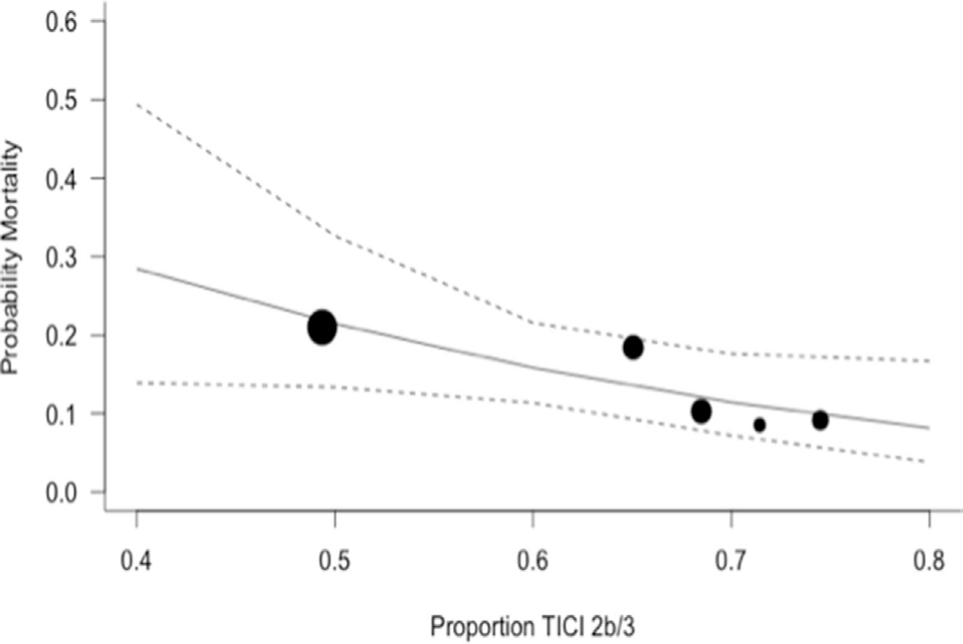
Predicted probability of death by modified thrombolysis in cerebral ischemia grade 2b/3, with dashed line showing 95% CI, and size of markers inversely proportional to each within-trial SD (intention-to-treat analysis).

The expected magnitude of clinical benefit associated with successful reperfusion can be shown in absolute terms by plotting the model predicted probability of good functional outcomes against the proportion of patients with mTICI 2b/3 (Figure 2). Every 10% increase in the rate of successful reperfusion over the range of 60–80% mTICI 2b/3 (corresponding to the rates reported in the trials) is associated with an 11% increase in the probability of good functional outcomes. The same increase in the rate of successful reperfusion is associated with a 17% increase in the rates of patients who recover with little to no symptoms (*Excellent outcomes*: mRS 0–1) and may reduce the rates of both SICH and death (Figures 2–5).

## Discussion

This meta-analysis of the stent-retriever-based acute stroke trials demonstrates strong evidence for an association between rates of successful reperfusion and good functional outcomes and very strong evidence for an association with excellent functional outcomes in early treatment windows. A large treatment effect based on increasing rates of successful reperfusion is suggested. Every 10% increase in the rate of successful reperfusion is associated with an 11% increase in the probability of achieving good functional outcomes and may be associated with reduced rates of death and symptomatic hemorrhage. Furthermore, there is a 17% increase in the rates of patients who recover with minimal or no symptoms (*excellent outcomes*).

The importance of reperfusion was established for IV-tPA in both the DEFUSE and EPITHET studies (21, 22). This was expanded upon with DEFUSE 2, which confirmed a large treatment effect for reperfusion in the setting of endovascular therapy. Moreover, the degree of reperfusion achieved in DEFUSE 2 was also highly significant. Increasing completeness of reperfusion being associated with increasing odds of good outcomes. Interestingly, subgroup analysis of DEFUSE 2 patients achieving reperfusion (>50% of at risk territory), showed no statistical difference in odds ratios for good outcomes in patients treated 0–6 versus 6–12 h from symptom onset (23). Recently, DEFUSE 3 and DAWN both achieved a significant treatment effect in patients treated >6 h from symptom onset, with both achieving high rates of successful reperfusion (8, 9). The current study adds to this body of evidence establishing the importance of successful reperfusion in the setting of modern endovascular stroke treatment.

Our analysis has several limitations; most obviously the reliance on trial level rather than patient level data, which due to the limited number of trials available for study, precludes the analysis of multiple predictors in the same model. In addition, as this is a *post hoc* meta-regression analysis of individual prospective trials, results can only be interpreted as associations rather than inferring causality.

Previously, stroke care systems have focused on reducing time metrics with the expectation that this will have the most dramatic impact on functional outcomes. It is likely, with the recent publication of extended time window endovascular trials (8, 9) that time limits will be extended. We suggest that rates of successful reperfusion will also need to be considered when designing stroke care systems and are likely to be even more significant in extended time windows.

## Conclusion

This analysis suggests reperfusion grade may be a signifcant modifable predictor of good functional outcomes for acute ischemic stroke of the anterior circulation. These results may have implications for stroke care network organization.

## Author Contributions

NM conceived and designed the study, collected data and drafted the manuscript. CW collected data, performed the statistical analysis, and contributed to manuscript drafting. PM provided guidance and oversight of the study and manuscript.

## Conflict of Interest Statement

CW is an employee of Roche Products Ltd. The views and opinions expressed in this paper are those of the author alone and do not necessarily reflect the views, policies, or any position of Roche Products Ltd. PM is a consultant for Stryker. The remaining author declares that the research was conducted in the absence of any commercial or financial relationships that could be construed as a potential conflict of interest.
