# Supplementary material for: Reperfusion and Clinical Outcomes in Acute Ischemic Stroke: Systematic Review and Meta-Analysis of the Stent-Retriever-Based, Early Window Endovascular Stroke Trials
Source: Front Neurol. 2018 May 14;9:301. doi: 10.3389/fneur.2018.00301 (PMC5968377; doi:10.3389/fneur.2018.00301)
Supplement: Supplementary file 1 [file data_sheet_1.DOCX]

**Supplementary tables for: Associations between rates of quality reperfusion with clinical outcomes: Systematic review and meta-analysis of the stent-retriever based endovascular trials.**

*Table 1.*

Full literature search on MEDLINE (via Ovid) for trials comparing stent-retriever thrombectomy to standard care in acute ischaemic stroke.

| **Search Number** | **Search Description** | **Number of Results** |
| --- | --- | --- |
| 1 | Brain ischaemia | 40094 |
| 2 | Randomized controlled trial | 409198 |
| 3 | Thrombectomy | 3887 |
| 4 | 1 AND 2 AND 3 | 8 |

*Table 2.*

Full literature search on EMBASE (via Ovid) for trials comparing stent-retriever thrombectomy to standard care in acute ischaemic stroke.

| **Search Number** | **Search Description** | **Number of Results** |
| --- | --- | --- |
| 1 | ‘Stroke’/exp OR stroke | 367288 |
| 2 | Randomized controlled trial | 493287 |
| 3 | Thrombectomy | 14,204 |
| 4 | 1 AND 2 AND 3 | 151 |

*Table 3.*

Summary of baseline patient characteristics by treatment group for the five included trials.

|  | **MR CLEAN** | | **REVASCAT** | | **ESCAPE** | | **SWIFT-PRIME** | | **EXTEND-IA** | |
| --- | --- | --- | --- | --- | --- | --- | --- | --- | --- | --- |
| Characteristics | Control  (N=267) | Intervention  (N=233) | Control  (N=103) | Intervention  (N=103) | Control  (N=150) | Intervention  (N=165) | Control  (N=98) | Intervention  (N=98) | Control  (N=35) | Intervention  (N=35) |
| Age,median (IQR) or mean (SD), years | 65·7 (55·5 - 76·4) | 65·8 (54·5 - 76·0) | 67·2 (9·5) | 65·7 (11·3) | 70·0 (60·0-81·0) | 71·0 (60·0-81·0) | 66·3 (11·3) | 65·0 (12·5) | 70·2 (11·8) | 68·6 (12·3) |
| Risk Factors, n (%) |  |  |  |  |  |  |  |  |  |  |
| Women | 110 (41·2) | 98 (42·0) | 49 (47·7) | 48 (46·6) | 79 (52·7) | 86 (52·1) | 51 (52·0)* | 44 (44·9) | 18 (51·4) | 18 (51·4) |
| Hypertension | 129 (48·3) | 98 (42·1) | 72 (69·9) | 62 (60·2) | 108 (72·0) | 105 (63·6) | 56 (57·7) | 66 (67·4) | 23 (65·7) | 21 (60·0) |
| Atrial fibrillation | 69 (25·8) | 66 (28·3) | 37 (35·9) | 35 (34·0) | 60 (40·0) | 61 (37·0) | 38 (39·2) | 35 (35·7) | 11 (31·4) | 12 (34·3) |
| Diabetes | 34 (12·7) | 34 (14·6) | 19 (18·4) | 22 (21·4) | 39 (26·0) | 33 (20·0) | 15 (15·5) | 12 (12·2) | 8 (22·9) | 2 (5·7) |
| Smoking | 78 (29·2)** | 65 (27·9)*** | 23 (22·5) | 26 (25·2) | 73 (49·3) | 80 (48·8) | 39 (39·8)^#^ | 41 (41·8)^##^ | 15 (42·9) | 12 (34·3) |
| NIHSS, median (IQR) | 18 (14-22) | 17 (14-21) | 17 (12-19) | 17 (14-20) | 17 (12-20) | 16 (13-20) | 17 (13-19) | 17 (13-20) | 13 (9-19) | 17 (13-20) |
| ASPECTS{Barber:2000th} | 9 (8-10) | 9 (7-10) | 8 (6-9) | 7 (6-9) | 9 (8-10) | 9 (8-10) | 9 (8-10) | 9 (7-10) | NA | NA |
| Large vessel occlusion |  |  |  |  |  |  |  |  |  |  |
| ICA | 78/(29·2)^###^ | 60 (25·8) | 28 (27·2)^¥^ | 26 (25·2)^###^ | 39 (26·0)^¥¥^ | 45 (27·3)^¥^ | 15 (15·3)^¥¥¥^ | 17 (17·3)^†^ | 11 (31·4) | 11 (31·4) |
| M1 MCA | 165 (61·8)^###^ | 154 (66·1) | 65 (63·1)^¥^ | 66 (64·1)^###^ | 105 (70·0)^¥¥^ | 111 (67·3)^¥^ | 72 (73·5)^¥¥¥^ | 62 (63·3)^†^ | 18 (51·4) | 20 (57·1) |
| M2 MCA | 21 (7·9)^###^ | 18 (7·7) | 8 (7·8)^¥^ | 10 (9·7)^###^ | 3 (2·0)^¥¥^ | 6 (3·6)^¥^ | 6 (6·1)^¥¥¥^ | 13 (13·3)^†^ | 6 (17·1) | 4 (11·4) |
| IV tPA, n (%) | 242 (91) | 203 (87) | 80 (77·7) | 70 (68·0) | 118 (78·7) | 120 (72·7) | 98 (100) | 98 (100) | 35 (100) | 35 (100) |

*Gender data not available in two patients.

**Smoking status not available in 15 patients.

***Smoking status not available in eight patients.

#Smoking status not available in five patients.

##Smoking status not available in two patients.

###Large vessel occlusion location not available in one patient.

¥Large vessel occlusion location not available in two patients.

¥¥Large vessel occlusion location not available in three patients.

¥¥¥Large vessel occlusion location not available in four patients.

†Large vessel occlusion location not available in five patients.

*Table 4.*

Summary of trial-level outcome data for the interventional group.

| **Trial** | **N_ITT*** | **mRS 0-2**  **(n/N_PP)**** | **mRS 0-1**  **(n/N_PP)**** | **Mortality**  **(n/N_PP)**** | **SICH**  **(n/N_PP)**** |
| --- | --- | --- | --- | --- | --- |
| **MR CLEAN** | 233 | 76/233 | 27/233 | 49/233 | 18/233 |
| **REVASCAT** | 103 | 45/103 | 25/103 | 19/103 | 2/103 |
| **ESCAPE** | 165 | 87/164 | 58/164 | 17/164 | 6/165 |
| **SWIFT-PRIME** | 98 | 59/98 | 42/98 | 9/98 | 0/98 |
| **EXTEND-IA** | 35 | 25/28 | 18/28 | 3/35 | 0/35 |
| **Total** | 634 | 287/626 | 173/626 | 97/633 | 26/634 |

* Number randomized to endovascular intervention (Intent-To-Treat)

** Denominator represents the number receiving intervention with data available (Per-Protocol)

*Table 5.*

Summary of reperfusion grades for the interventional population across the five trails.

| **Study**  **(n)** | **%mTICI 0 (n)** | **%mTICI 1 (n)** | **%mTICI 2a (n)** | **%mTICI 2b (n)** | **%mTICI 3 (n)** | **%mTICI 2b/3 (n)** |
| --- | --- | --- | --- | --- | --- | --- |
| **MR CLEAN (196)** | 13·8 (27) | 5·6 (11) | 21·9 (43) | 34·7 (68) | 24·0 (47) | 58·7 (115) |
| **REVASCAT (102)** | 7·8 (8) | 2·0 (2) | 24·5 (25) | 47·1 (48) | 18·6 (19) | 65·7 (67) |
| **ESCAPE (156)** | N/A | N/A | N/A | N/A | N/A | 72·4 (113) |
| **SWIFT-PRIME (83)** | 4·8 (4) | 1·2 (1) | 6·0 (5) | 19·3 (16) | 68·7 (57) | 88·0 (73) |
| **EXTEND-IA (29)** | 3·4 (1) | 3·4 (1) | 6·9 (2) | 37·9 (11) | 48·3 (14) | 86·2 (25) |

*Table 6.*

Unadjusted odds ratios for associations between 10% increase in rate of quality reperfusion (mTICI 2b/3) and outcomes in the intervention population.

| **Outcome** | **OR** | **95% CI** | **P-value** |
| --- | --- | --- | --- |
| Good functional outcome (mRS 0 -2) | 1.59 | (1.16, 2.19) | 0.019 |
| Excellent functional  Outcome (mRS 0-1) | 2.10 | (1.46, 3.01) | 0.007 |
| Mortality | 0.69 | (0.47, 1.01) | 0.053 |
| SICH | 0.54 | (0.28, 1.04) | 0.057 |

Based on ITT analysis.

Figure 1.


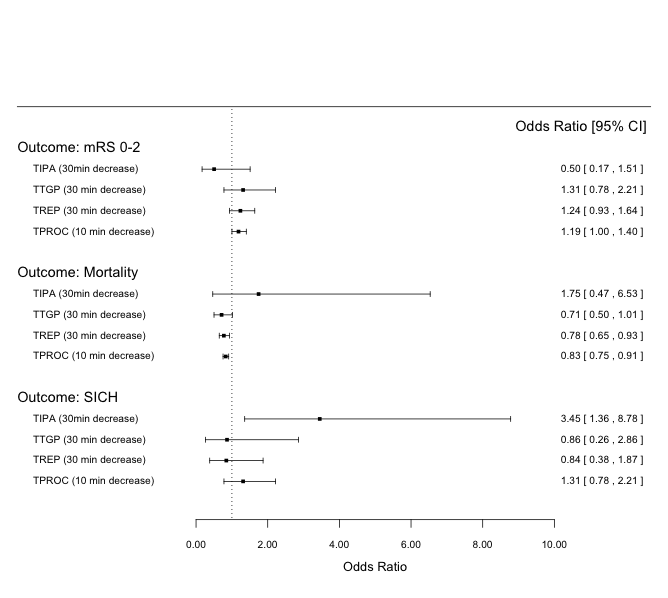


Forest plots showing odds ratios for association between time metrics and patient outcomes. TIPA = time to IV-tPA, TTGP = time to groin puncture, TREP = time to reperfusion, TPROC = procedure time.

Figure 2.


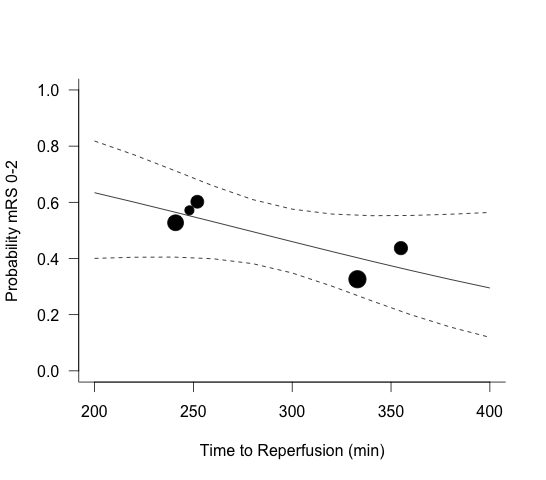


Predicted probability of mRS 0-2 by time to reperfusion, with dash line showing 95% CI, and size of markers inversely proportional to each within-trial standard deviation (ITT analysis).
