# Supplementary material for: Reperfusion and Clinical Outcomes in Acute Ischemic Stroke: Systematic Review and Meta-Analysis of the Stent-Retriever-Based, Early Window Endovascular Stroke Trials
Source: Front Neurol. 2018 May 14;9:301. doi: 10.3389/fneur.2018.00301 (PMC5968377; doi:10.3389/fneur.2018.00301)
Supplement: Supplementary file 2 [file data_sheet_2.DOC]

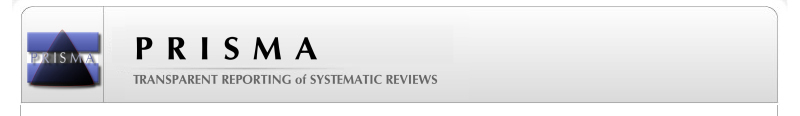
**PRISMA Flow Diagram**

**Screening**

**Included**

**Eligibility**

**Identification**

Records identified through database searching
(n = 159)

Additional records identified through other sources
(n = 0)

Records after duplicates removed
(n =151)

Records screened
(n =151)

Records excluded
(n = 143)

Full-text articles assessed for eligibility
(n = 8)

Full-text articles excluded, with reasons: <75% stent-retriever use per ITT
(THERAPY, THRACE, PISTE)

Studies included in qualitative synthesis
(n = 5)

Studies included in quantitative synthesis (meta-analysis)
(n =5)
